# Cardoon Meal as Alternative Protein Source to Soybean Meal for Limousine Bulls Fattening Period: Effects on Growth Performances and Meat Quality Traits

**DOI:** 10.3390/ani11123383

**Published:** 2021-11-26

**Authors:** Lapo Nannucci, Francesco Mariottini, Silvia Parrini, Francesco Sirtori, Riccardo Bozzi, Michele Falce, Chiara Aquilani, Andrea Confessore, Antonello Cannas, Giovanni Brajon

**Affiliations:** 1 Istituto Zooprofilattico Sperimentale del Lazio e della Toscana ‘M. Aleandri’, Via Castelpulci 43, 50018 Florence, Italy; lapo.nannucci@gmail.com (L.N.); francescomariottini93@gmail.com (F.M.); giovanni.brajon@izslt.it (G.B.); 2 Dipartimento di Scienze e Tecnologie Agrarie Alimentari Ambientali e Forestali, Università degli Studi di Firenze, Piazzale delle Cascine 18, 50144 Florence, Italy; francesco.sirtori@unifi.it (F.S.); riccardo.bozzi@unifi.it (R.B.); chiara.aquilani@unifi.it (C.A.); andrea.confessore@unifi.it (A.C.); 3 Novamont Spa, Via G. Fauser 8, 28100 Novara, Italy; michele.falce@novamont.com; 4 Dipartimento di Agraria, Università degli Studi di Sassari, Viale Italia 39, 07100 Sassari, Italy; cannas@uniss.it

**Keywords:** beef cattle, by-product, *Cynara cardunculus*, sustainable animal production

## Abstract

**Simple Summary:**

Beef cattle feeding strategies are commonly based on soybean meal utilization as a fundamental protein source. This feed, though, might have negative environmental impacts on the major areas of production and is becoming very expensive. Cardoon (*Cynara cardunculus* L.) is a resilient crop which represents a good opportunity in reclaiming and remediating unutilized lands. Cardoon seeds are rich in oil, which is extracted for industrial purposes, and the related by-products (press cake and meal) are characterized by high protein content and essential fatty acids. The aim of this study was to evaluate cardoon meal as a protein source during the Limousine bulls’ fattening period, in order to study a suitable alternative to develop and create low-input and low-emission feeding strategies. The results obtained in terms of growth performances showed no statistical difference between bulls fed the by control diet (containing soybean meal as main protein source) and animals fed by the experimental diet, where soybean meal was partially replaced by one containing cardoon meal. Meat quality traits were measured, and no differences between the groups were found. Hence, these by-products could be considered as a valuable solution in Limousine bulls’ fattening periods and could be used to represent a key factor to improve cattle-feeding sustainability.

**Abstract:**

Soybean meal is the most important protein source in beef cattle feeding. The research of alternative protein sources to replace soy use, avoiding negative effects on in vivo performance and on the product’s quality, is an important issue. In this context, cardoon represents a non-OGM resilient crop that can be cultivated in marginal lands for extracting its seed oil (utilized for biodiesel and biodegradable bioplastic production) and whose and the residual meal from its seed oil (utilized for biodiesel and biodegradable bioplastic production) could be a suitable by-product for animal feeding, due to its fairly high protein content. The aim of this study was to evaluate the feasibility of using cardoon meal as an innovative protein source during the Limousine bulls’ fattening period. Thirty-two bulls were divided into two groups and fed with a diet containing soybean meal (SG) or partially replacing soybean meal with cardoon meal as a protein source (CG), respectively. The feeding trial lasted about 11 months. Growth performances and meat physical–chemical traits were evaluated. No statistical differences in feed efficiency, average daily gain, or in the main meat quality indicators, as well as in fatty acid profiles were found among the groups. Therefore, cardoon meal could be considered as an alternative to soybean meal in fattening Limousine bulls in order to enhance the sustainability of the farming system.

## 1. Introduction

According to the Food and Agriculture Organization, the world’s population will reach 9.6 billion people by 2050, and by consequence, the request for high-value protein sources will increase. Global food systems will therefore need to meet the dietary demands of more people, who, on average, will be wealthier than people of today and will aspire to food choices that are currently only available in high-income countries [1]. Among the types of food expected to be involved by this phenomenon, there will be products of animal origin. These products will also have to consider the new orientations of consumers’ thinking towards farms that respect animal welfare. Today, animal welfare takes on a wider meaning than in the past (also defined as an interdisciplinary scientific field) considering aspects such as behavior, but also social and ethical attributes [2,3,4].

Furthermore, future beef production will have to address environmental sustainability issues, striving to reduce greenhouse gas (GHG) emissions and to cope with climate change, showing proficiency in keeping up with the best drivers in terms of environmental challenges [5]. In this context, the main protein source for beef cattle feeding is soybean meal, because it is widely available and richer in valuable rumen undegradable protein (RUP) than most other protein sources. However, most soy is imported from long distances and its cultivation is increasingly debated due to its putative negative environmental impacts (e.g., monocropping, long transport routes, loss of biodiversity and natural habitats, and high-input requirements, such as fertilizers, land, and fuel) and poor socioeconomics (e.g., rural depopulation, high levels of mechanization and agrarian transformation, and related loss of human employment) [6,7]. The areas cultivated with soy are still increasing at a global level, and there are many Life Cycle Assessments (LCAs) focusing on the environmental impacts of soybean cultivation [8]. Due to the high costs of soy, beef producers have been seeking an alternative protein source that could reduce feeding costs [9]. Dietary utilization of plant by-products is a suitable approach for sustaining low-emission and low-input feeding strategies to reduce the environmental impact of livestock production [10,11]. Animal production sustainability is affected by food vs. feed competition under several aspects, such as employment of human-edible crops, impact of mechanical soil tillage, and soil and water exploitation. The introduction of a resilient crop as an alternative protein source could contribute to mitigate the above-mentioned constraints and, therefore, improve the use of marginal lands. Cardoon (*Cynara cardunculus* L.) is a perennial species that originates in the Mediterranean basin. It is a resilient rainfed crop that also yields under water and weed stress conditions [12,13], and on terrains that are not exploitable by intensive high-input and water-eager agriculture, reducing the competition for land use between food and no-food crops [14,15]. Moreover, it represents a good opportunity to recover and exploit marginal and unutilized lands [16]. The oil extracted by cardoon (*Cynara cardunculus* L. var. altilis) seed is mainly used for biodiesel and biodegradable plastic production [17]. The by-products obtained through oil extraction, such as seed-press cake and meal, could be considered due to their chemical characteristics as a potential source of protein and bioactive molecules to be used in animal feeding. Up until now, cardoon forage and meal have been used in small ruminant feeding due to their high fiber content (approximately 58% in neutral detergent fiber (NDF)) and also thanks to the rumen microbiota efficiency in catabolizing cellulose [18,19]. The use of by-products from the production of biodiesel and bioethanol (distillate based on wheat and barley dry cereals and rapeseed meal) on the performance of growing fattening bulls has already been reported [20], while no information about cardoon by-products’ inclusion in beef cattle feeding and its related effects in meat quality is currently available. Due to its high content in protein and essential fatty acids, it could be interesting to test the partial substitution of meal (SM) with cardoon meal (CM) in beef cattle feeding [21].

The aim of this study was to evaluate cardoon meal as a replacement of soybean meal in the diet of Limousine bulls during their fattening period in order to verify the effects on growth performance and meat quality traits.

## 2. Materials and Methods

### 2.1. Experimental Design

#### 2.1.1. Animals, Diets, and Management

Thirty-two purebred Limousine bulls reared in a Tuscan farm located in Mugello, Italy were selected by considering their age (about 300 days ± 19.97 as SD) and raised indoors during the fattening period. Animals were kept in four boxes of 5 m × 5.2 m with permanent straw litter (two boxes for each diet and eight animals per box) and individually identified by ear tag. A monofactorial experimental design with two levels (diets) was used. The experimental unit was the animal, except for the DM intake, since the animals were group-fed. At the beginning of the trial, animals were randomly assigned to one of two diets: group 1 (SG) was fed with the conventional diet used by the farm containing SM as the more relevant protein source; and in group 2 (CG) the SM was partially replaced by CM. The chemical composition of cardoon meal and soybean meal used in the trial is reported in Table 1, while the composition of the two diets is reported in Table 2.

The average weight of animals at the beginning of the trial was about 400 kg ± 45.61.

The feeding was administered once a day by a total mixed ration wagon. The vehicle was equipped with a software to calculate the amount of each component of the diet in order to meet the animal’s daily nutritional requirements. Daily feed amounts were progressively increased according to the bulls’ growth, starting from about 10 kg/d DM per animal at the beginning of the trial, to about 14 kg/d DM per animal at the end of the fattening period. The water (from a controlled natural source) was available ad libitum thanks to the presence of two drinking bowls within each box.

Every month, the bulls were individually weighed. The average feed intake per animal was calculated by dividing the total amount of feed consumed per box by the overall number of animals kept in the same box. Feed efficiency was calculated for each group as the estimated individual feed intake/individual weight gain ratio. On average, the feed intake was 13.26 kg/d of DM for CG and 13.21 kg/d o/DM for SG. The animals were slaughtered after an average fattening period of 11 months ± 1.18 from the beginning of the trial, when they were about 21 months old. The average slaughter weight was 779 ± 80.2 kg.

#### 2.1.2. Animal Health Welfare Evaluation

Animal welfare assessment on the farm was performed through the use of the Classyfarm system checklist for beef cattle (Classyfarm.it). At the start of the trial, sanitary controls were conducted on the bulls to ensure the good health condition of the animals and the absence of contiguous infectious or parasitic disease. Fecal samples were collected directly from the rectum of each animal and placed into plastic cups. Samples were then transported to the laboratory where they were immediately stored at 4 °C and processed the day after. Feces were analyzed by the flotation technique for the research of enteroparasite eggs (roundworms and tapeworms), while an immunofluorescence test was carried out to detect Giardia duodenalis and Cryptosporidium parvum. Fecal samples were also analyzed through real-time PCR for the Bovine Coronavirus and Bovine Rotavirus.

#### 2.1.3. Diet Chemical Analysis

Diets were analyzed to evaluate the proximate profile, as reported in Table 3. Determined parameters were: dry matter (DM), crude protein (CP), crude fat (CF), crude fiber (CF), and ash. They were assessed according to the AOAC methods [22]. Neutral detergent fiber (NDF), acid detergent fiber (ADF), and acid detergent lignin (ADL) were determined according to van Soest et al. [23], using heat stable amylase and sodium sulphite, and expressed inclusive of residual ash. Metabolizable energy (ME) was estimated from feed tables according to Sauvant et al. [24].

Fatty acids of SG and CG diets were analyzed using a modified method by Folch et al. [25] for the lipid extract and a Varian GC-430 apparatus equipped with a flame ionization detector (FID) (Palo Alto, CA, USA) for the fatty acid profile determination. The individual methyl esters were identified by their retention time using an analytical standard (F.A.M.E. Mix, C8-C22 Supelco 18,920-1AMP). Results based on the internal standard (C23:0) were expressed as the percentage of total fatty acids (Table 4).

#### 2.1.4. Carcass Traits’ Physical and Chemical Analysis

Bulls were slaughtered in an authorized commercial EU-licensed abattoir. Animals were transported to the abattoir directly from the farm and the transport and slaughtering conditions were the same for all the animals.

After 24 h from the slaughter, the carcasses were quartered into fore- and hindquarters and a meat sample (T-bone) from the first hindquarter vertebra was taken from each animal. From each meat sample, Longissimus dorsi (LD) was collected to perform physical and chemical analysis. Color parameters CIE L* (lightness), a* (redness), and b* (yellowness) were measured on LD meat slices (1 cm thick) using a Minolta colorimeter CR-200 (Minolta Camera Co., Ltd., Osaka, Japan). The top of the Chroma Meter measuring head was placed in a flat position against the meat’s surface. The reflective color was determined from the average of three consecutive pulses from the optical chamber of the colorimeter. For each sample, three measurements were performed. Data are reported in the L* a* b* color notation system [26] with L* axis representing lightness, the a* axis representing the green-red color axis (redness) and the b* axis representing the blue-yellow (yellowness) color axis. Three raw meat cubes (1 cm × 1 cm × 1 cm) were obtained from each LD sample to determine texture profile analysis (TPA) analysis using a Zwick Roell Z2.5 apparatus (Ulm, Germany) with a loading cell of 1 kN at the crosshead speed of 1 mm/s. TPA curve-forces were determined by a compression plate of 100 mm diameter. Samples were axially compressed to 75% of the original height. TPA parameters, hardness, springiness, and cohesiveness were recorded, while chewiness was calculated.

Moisture (through lyophilization to constant weight), total protein, and ash content were determined following the AOAC methods [22] on minced portions of LD. On the same sample, the total lipid content was analyzed as described by Folch et al. [25]. The fatty acids profile was determined on the lipid extract using a Varian GC-430 apparatus equipped with a flame ionization detector (FID) (Palo Alto, CA, USA). FA separation occurred in a Supelco Omegawax TM 320 capillary column (30 m length; 0.32 mm internal diameter; 0.25 µm film thickness; Supelco, Bellafonte, PA, USA). The chromatographic conditions were an initial temperature of 160 °C, which was then increased by 2 °C/min until reaching 220 °C. A total of 1 µL of the hexane-diluted sample was injected with the carrier gas (helium) at a constant flow of 1.5 mL/min and at a split ratio of 1:20. The detector temperature was set at 260 °C. The individual methyl esters identification was made by their retention time using an analytical standard (F.A.M.E. Mix, C8-C22 Supelco 18,920-1AMP). Response factors based on the internal standard (C23:0) were used for quantification, and results were expressed as g/100 g on a wet basis.

#### 2.1.5. Statistical Analysis

Data were analyzed with the SAS software package using the GLM procedure with the following model:

Y_ijk_ = μ + D_i_ + B_j_ + b(X_ijk_) + ε_ijk_,

where Y = depend variable; μ = mean; D = Diet; B = Box; b = regression coefficient; X = independent variable (days of trial); and ε = random error effect.

The Student’s t-test was used to test the differences between least square means. The statistical significance was established at *p* < 0.05.

## 3. Results

### 3.1. Growth Performance

The animals’ growth performances are presented in Table 5. The partial substitution of soybean meal with cardoon meal did not appear to have affected in vivo performances: age at the start of the trial was about 300 days, and the initial weight was about 400 kg. The average daily gain (ADG) was the same for each diet group (about 1.1 kg per day). The final weight of the groups was about 779 kg.

### 3.2. Animal Health and Welfare Evaluation

Sanitary controls showed that all the animals were in a good health condition. The bulls were free of enteroparasites, *Giardia duodenalis* and *Cryptosporidium parvum*, and the Bovine Coronavirus and Bovine Rotavirus. The farm joined the regional eradication plan for infectious bovine rhinotracheitis (IBR) and has been recognized as officially free. The farm is also officially free from brucellosis and bovine leukosis. All animals included in the study were vaccinated for the bovine syncytial virus, parainfluence virus, *Mannheimia haemolytic*, and clostridiosis. No deaths and no animal exclusion occurred during the trial.

### 3.3. Meat Quality Traits

All carcasses were evaluated for the dressing and major traits through the SEUROP System scoring U2 (30 animals) and U3 (two animals). Results of meat quality traits are shown in Table 6. Comparing the dietary groups, no significant differences were found among treatments both according to chemical and physical characteristics. TPA parameters did not show differences between the two experimental groups.

The fatty acid analysis of meat did not show differences between the two groups. The partial substitution of SM with CM neither affected the percentage of fatty acid groups, such as saturated fatty acids (SFA), monounsaturated fatty acids (MUFA), polyunsaturated fatty acids (PUFA), nor the single fatty acids (Table 7).

## 4. Discussion

The lack of significant differences between the two groups in terms of final weight and ADG indicated that the replacement of 50% SM with CM did not affect animal growth performances. Salami et al. [18] reported on the use of CM in lambs, replacing 15% of dehydrated alfalfa for 75 days, highlighting no differences due to the treatments in terms of final weight and ADG. Considering that our trial lasted for the entire fattening period (11 months), this scenario could probably suggest that the replacement of different protein sources with CM does not affect growth performances independently from the duration of the feeding period. On the other hand, also in some studies such as that by Kurrig et al. [27], the replacement of soybean meal with other alternative protein sources (faba beans, pumpkin seed, and spirulina) in Limousine bulls for about 500 days did not affect the animals’ performance, such as growth in body weight and average daily gain.

The replacement of SM with CM in our study did not affect any meat chemical parameters, as no differences were found between the SG and CG samples. Thus, in our study, meat quality was not affected by the protein sources of the diets. Similar results have been observed testing the replacement of SM with other types of by-products. Meyer et al. [20] reported that by-product protein sources did not influence the quality of carcasses in Holstein bulls. Segers et al. [28] used dried distilled grains and corn gluten in Angus beef, and found that the diet did not lead to differences in terms of meat protein and lipid content between animals that were fed soybean diets and those that were fed alternative protein sources.

On the other hand, the inclusion of CM also did not affect meat chemical composition in poultry, as reported by Buccioni et al. [29], where this feed component was tested on the finishing period of Kabir chickens for about 30 days. In this case, SM was partially (16%) or completely replaced with CM.

Besides meat quality chemical traits, for physical parameters also, no differences between the two theses were observed. With regard to texture, it is important to consider that the bulls were under the same rearing system and climate conditions, and they also had the same available space, breed, age, and gender. Those factors play important effects on texture, whereas diet seems to make a minor contribution to the tenderness of meat [30]. Several studies reported physical exercise as one determinant factor of meat texture characteristics. Indeed, Aalhus and Price [31] concluded that exercise was the main factor that can influence the type, size, and composition of muscle fibre and, therefore, meat texture. However, in our study, the bulls of two dietary groups were subjected to the same physical conditions and activity.

For hardness and chewiness, the lack of a difference could also be related to the same intramuscular fat content between groups, that represents a parameter strictly connected to these two parameters according to Sasaki et al. [32].

The partial replacement with the CM did not bring about any changes even in the color parameters of meat, according to the results obtained by Salami et al. [18] on lamb meat, where CM was replaced with dehydrated alfafa. In addition, the use of other by-products seemed to not affect the meat color. In a feeding trial for lambs on the use of pumpkin seed cake to partially replace SM in 70 days, Antunović et al. [33] reported that there were not any changes in meat color. Considering that the animals in our trial had almost the same age and that any differences in intramuscular fat content were spotted between the two groups, these results are in line with the above-mentioned studies. We could also assume that the partial substitution of SM with CM on ruminant diets is not an influencing factor for meat color parameters’ variability according to Salami et al. [18], who suggested that neither the CM diet, nor the interaction between diet and time affected the color stability of raw lamb meat.

The replacement of SM with CM did not affect the meat fatty acids profile, and thus the CG meat had the same qualitative level as SG meat. This last result should be considered as one of the most important responses of our trial, because it suggests that CM could represent a sustainable innovative protein source to replace SM.

In fact, according to the results on lamb meat fatty acids profiles reported by Salami et al., dietary treatment did not influence the total lipids nor the relative composition of SFA, MUFA, and PUFA [18]. In this latter study, CM did not affect the intramuscular composition in terms of different fatty acid families, but presented a lower concentration of potentially health-promoting fatty acids (vaccenic and rumenic acids) in lamb meat. The utilization of various alternative protein sources for the substitution of SM in small and large ruminant diets seemed to have moderate effects on the meat fatty acids profile, reported by various studies. The replacement of SM in diets was tested by Keller et al. [34], who reported the effects of the inclusion of faba beans, pumpkin seed cake, and spirulina as protein sources in the diets of Limousine-sired crossbred bulls on the intramuscular fat quality. Few changes in the fatty acids profile (C16:0 iso, C18:1 cis-12, and, in tendency, in C18:1 trans-11) of the intramuscular fat were observed. On the contrary, Neto et al. [35] reported that replacement of ground soybean with 24% DM of ground cotton seed in the Red Norte bulls’ fattening diet showed an increase in terms of the saturated fatty acids content of the LD. Nevertheless, according to our results, it seems that CM did not have an effect both on the fatty acids profile and on the oxidative stability of meat [18], even if it is considered a rich source of polyphenols with strong antioxidant activity.

To date, there is no evidence of feeding trials based on the dietary inclusion of cardoon by-products for Limousine bulls in the literature. Nevertheless, cardoon meal also represents a sustainable solution for farmers in terms of direct costs. The market value of cardoon meal is established using sunflower meal as a reference, as these products have similar characteristics. Therefore, at the time of the trial, the related price per ton was about €165, while soybean meal in the same period showed a price of about €334 euros per ton [36]. In this context, cardoon by-products can represent not only an important feed source option, but also a valuable example of integration between green chemistry, the industry, and traditional farming [37], while simultaneously increasing the sustainability of beef cattle production.

## 5. Conclusions

The current study demonstrated that CM could be considered as a valuable alternative to partially replace SM in Limousine bulls during their fattening period (11 months). In fact, no statistical differences in terms of growth performance (final weight and individual average daily gain) were found among the two groups. In addition, the meat quality (LD physical and chemical analysis) and fatty acids profile did not differ between the two dietary groups.

Thus, it appears that cardoon meal can be used to partially substitute soybean meal., If compared to soybean meal, it also has advantages in terms of environmental impact and crop resilience. Thus, it can be considered as a valuable protein source in improving sustainable livestock feeding, especially in marginal areas where intensive high-input crops are not suitable, suggesting that it is possible to produce a valuable protein source at local level, reducing the amount of protein supplement produced overseas and then imported at high environmental and financial cost. Considering that cardoon meal is a by-product of oil extraction for bioplastic production, it could play an active role in increasing the sustainability of beef cattle production systems. Further investigations are needed to deepen the knowledge of nutritional effects of cardoon meal in ruminants, both to identify different feeding formulations with increasing levels of replacement and to implement the current knowledge on animal growth performances and meat quality traits.

## Figures and Tables

**Table 1:** Cardoon meal and soybean meal chemical composition (g/100 g DM).

Nutritional Component	Cardoon Meal	Soybean Meal
Dry matter, g/100 g as fed	87.78	89.34
Crude Protein	21.33	48.58
Crude fat	1.02	1.00
Ash	6.38	7.81
NDF	58.71	17.39
ADF	52.00	13.13
ADL	24.99	7.23

NDF = Neutral detergent fibre; ADF = Acid detergent fibre; ADL = Acid detergent lignin.

**Table 2:** Diet composition (% as DM) of the soybean meal group (SG) and cardoon meal group (CG).

Ingredients	CG ^1^	SG ^1^
Mixed hay	8.13	8.48
Alfa alfa hay	11.26	11.75
Maize silage	22.58	23.55
Wheat middlings	12.62	13.16
Maize meal	20.07	20.93
Rolled barley	12.02	12.54
Field bean	1.38	1.44
Soybean meal	3.24	6.74
Cardoon meal	7.34	-
Bicarbonate Sodium	1.36	1.41

^1^ Group 1 (SG) was fed a diet with soybean meal and without cardoon meal; in group 2 (CG) 50% of soybean meal was replaced by cardoon meal.

**Table 3:** Chemical composition (g/100 g DM) and nutritional value (Mcal/kg of DM) of the diets.

Nutritional Component	CG ^1^	SG ^1^
Dry matter (DM), g/100 g as fed	64.00	63.00
Crude Protein	12.50	13.20
Crude fat	4.46	5.02
NDF	36.90	35.30
ADF	26.93	6.28
ADL	11.48	1.94
Ca	0.31	0.30
P	0.46	0.44
Metabolizable energy	2.19	2.31

^1^ Group 1 (SG) was fed a diet with soybean meal and without cardoon meal; in group 2 (CG) 50% of soybean meal was replaced by cardoon meal. NDF = Neutral detergent fibre; ADF = Acid detergent fibre; ADL = Acid detergent lignin; DM = Dry matter.

**Table 4:** Fatty acids profile of soybean meal and cardoon meal (percentage of total fatty acids).

Fatty Acid	Abbreviation	Cardoon Meal	Soybean Meal
Lauric acid	C12:0	0.10	0.04
Myiristic acid	C14:0	0.21	0.18
Palmitic acid	C16:0	11.82	15.18
Palmitoleic acid	C16:1	0.40	0.28
Stearic acid	C18:0	4.65	4.97
Oleic acid	C18:1	27.79	15.44
Linoleic acid	C18:2 n6	49.58	53.56
Linolenic acid	C18:3 n3	0.72	7.86
Arachidic acid	C20:0	0.66	0.26
Eicosenoic acid	C20:1 n9	0.26	0.20
Behenic acid	C22:0	0.57	0.51
Saturated Fatty acids	SFA	19.00	21.77
Monounsaturated fatty acids	MUFA	29.52	16.32
Poliunsaturated fatty acids n6	PUFA 6	50.37	53.73
Poliunsaturated fatty acids n3	PUFA 3	0.72	8.04
Poliunsaturated fatty acids	PUFA	51.09	61.77

**Table 5:** Growth performances.

Item	CG ^1^	SG ^1^	SEM	*p*-Value
Initial Age (d)	304	303	5.248	0.936
Initial weight (kg)	401.93	399.81	13.850	0.914
Final weight (kg)	779.07	778.93	10.608	0.993
Average daily gain (kg)	1.148	1.159	0.031	0.809
Feed conversion ratio (kg of feed DM/kg of BW gain)	11.55	11.4	0.34	0.756

^1^ Group 1 (SG) fed a diet with soybean meal and without cardoon meal; group 2 (CG) in which 50% of soybean meal was replaced by cardoon meal. *p*-value, Probability of significant effect due to experimental factors (threshold *p* < 0.05); SEM, standard error of the mean.

**Table 6:** Meat chemical and physical traits.

Item	CG ^1^	SG ^1^	SEM	*p*-Value
Moisture %	71.662	71.818	0.369	0.773
Lipids %	2.588	2.512	0.323	0.872
Total Proteins %	24.407	24.315	0.280	0.822
Ash %	1.340	1.353	0.042	0.838
Cohesivity	0.250	0.250	0.010	0.988
Hardness (N)	54.211	61.075	5.863	0.425
Springiness (mm)	1.816	1.769	0.066	0.629
Chewiness (N × mm)	24.649	28.299	3.302	0.437
L*	43.442	45.239	0.683	0.080
a*	16.882	17.635	0.621	0.409
b*	3.191	3.884	0.733	0.518

^1^ Group 1 (SG) fed a diet with soybean meal and without cardoon meal; group 2 (CG) in which 50% of soybean meal was replaced by cardoon meal. *p*-value, Probability of significant effect due to experimental factors (threshold *p* < 0.05); SEM, standard error of the mean.

**Table 7:** Fatty acids profile (% of total fatty acids) of the meat.

Fatty Acid		CG ^1^	SG ^1^	SEM	*p*-Value
Lauric acid	C12:0	0.03	0.04	0.002	0.363
Myiristic acid	C14:0	2.19	2.14	0.125	0.792
Pentadecylic acid	C15:0	0.36	0.35	0.265	0.823
Palmitic acid	C16:0	26.36	27.36	0.638	0.287
Palmitoleic acid	C16:1	2.11	2.08	0.110	0.856
Heptadecanoic acid	C17:0	0.91	0.91	0.424	0.957
Heptadecenoic acid	C17:1	0.37	0.39	0.208	0.596
Stearic acid	C18:0	19.36	20.33	0.633	0.296
Oleic acid	C18:1	30.96	31.09	0.903	0.922
Linoleic acid	C18:2 n6	10.99	9.49	1.054	0.335
Linolenic acid	C18:3 n3	0.53	0.51	0.326	0.612
Arachidic acid	C20:0	0.1	0.11	0.005	0.214
Eicosenoic acid	C20:1 n9	0.09	0.09	0.006	0.745
Eicosadienoic acid	C20:2 n6	0.08	0.09	0.009	0.286
Dihomo-γ-linolenic acid	C20:3 n6	0.53	0.63	0.730	0.354
Arachidonic acid	C20:4 n6	2.03	2.48	0.300	0.372
Eicosatrienoic acid	C20:3 n3	0.01	0.02	0.002	0.110
Eicosatetraenoic acid	C20:4 n3	0.03	0.03	0.002	0.375
Eicosapentaenoic acid	C20:5 n3	0.10	0.12	0.142	0.353
Behenic acid	C22:0	0.04	0.04	0.004	0.656
Erucic acid	C22:1 n9	0.16	0.16	0.001	0.947
Adrenic acid	C22:4 n6	0.19	0.23	0.279	0.336
Docosapentaenoic acid	C22:5 n6	0.02	0.02	0.003	0.67
Docosapentaenoic acid (DPA)	C22:5 n3	0.26	0.31	0.035	0.328
Docosahexaenoic acid	C22:6 n3	0.03	0.03	0.004	0.190
Saturated Fatty acids	SFA	49.99	52.55	1.143	0.134
Monounsaturated fatty acids	MUFA	33.87	34.00	1.018	0.930
Poliunsaturated fatty acids n6	PUFA 6	14.86	12.3	1.400	0.218
Poliunsaturated fatty acids n3	PUFA 3	1.23	1.09	0.199	0.073
Poliunsaturated fatty acids	PUFA	16.14	13.45	1.469	0.216

^1^ Group 1 (SG) fed a diet with soybean meal and without cardoon meal; group 2 (CG) in which 50% of soybean meal was replaced by cardoon meal. *p*-value, Probability of significant effect due to experimental factors (threshold *p* < 0.05); SEM, standard error of the mean.

## Data Availability

Not applicable.
